# Comparison of high-flow nasal cannula oxygen therapy versus nasal cannula in sedated transoesophageal echocardiography in patients with mitral regurgitation: a prospective, randomized controlled clinical trial

**DOI:** 10.1186/s13019-026-04044-6

**Published:** 2026-04-18

**Authors:** Jing Hu, Ziqi Qiu, Daolin Xia, Wenwen Zhang, Zhaojing Fang, Meirong Ling, Mingxia Ding

**Affiliations:** 1 Department of Anesthesiology, Perioperative and Pain Medicine, Nanjing First Hospital, Nanjing Medical University, Nanjing, China; 2 Department of Anesthesiology, Xuyi People’s Hospital, Kangda College of Nanjing Medical University, Huaian, Jiangsu China; 3 Emergency Medical Department, Minhang Hospital, Fudan University, Shanghai, China; 4 Department of Anesthesiology, Wuxi No.2 People’s Hospital, Wuxi Clinical College of Nantong University, Wuxi, Jiangsu China

**Keywords:** High flow nasal cannula, Transoesophageal echocardiography, Sedation, Hypoxia

## Abstract

**Background:**

Patients with moderate-to-severe mitral regurgitation (MR) undergoing pre-procedural transesophageal echocardiography (TEE) for transcatheter edge-to-edge repair (TEER) are at high risk for hypoxia during propofol sedation. We evaluated the efficacy and safety of high-flow nasal cannula (HFNC) oxygen therapy compared with conventional nasal cannula in this population.

**Methods:**

This prospective, single-center, randomized controlled trial enrolled 263 patients with moderate-to-severe MR scheduled for pre-procedural TEE under propofol sedation. Patients were randomized to receive either HFNC or conventional nasal cannula. The primary outcome was the incidence of hypoxia. Secondary outcomes included subclinical respiratory depression, need for airway interventions, hemodynamic parameters, adverse events, and satisfaction scores.

**Results:**

HFNC significantly reduced hypoxia incidence compared with conventional oxygen (13.74% vs. 23.48%; relative risk 0.59, 95% CI 0.35–0.99; *p* = 0.040). HFNC also decreased subclinical respiratory depression (18.32% vs. 32.58%; *p* = 0.007) and need for jaw-lift maneuvers (20.61% vs. 33.33%; *p* = 0.026). No patients in the HFNC group required mask ventilation versus 12.88% in the control group (*p* < 0.001). Operator and anesthesiologist satisfaction scores were higher with HFNC (both *p* < 0.01). No serious HFNC-related adverse events occurred.

**Conclusions:**

HFNC significantly reduces hypoxia and airway interventions during pre-procedural TEE in patients with MR, with improved safety and satisfaction profiles.

## Introduction

Mitral regurgitation (MR) is the most common valvular heart disease [1]. In China, the number of individuals with grade III or higher MR exceeds 10 million, necessitating therapeutic interventions [2, 3]. Surgical valve repair or replacement is considered the standard method for treating MR and has been proven to relieve patient symptoms and prolong survival. However, 50% of patients with MR do not receive effective treatment because of high-risk factors such as poor cardiac function, multiple comorbidities, and advanced age, which makes them unsuitable for surgery [4]. For patients who are considered too elderly, too ill, or too high-risk for conventional open-heart surgery, the MitraClip offers a viable therapeutic option [5].Before undergoing Transcatheter edge-to-edge repair (TEER) procedures, echocardiography is a crucial technique for patient selection, as it helps assess cardiac and valve function according to the EVEREST criteria [6].

To ease patients’ apprehension and fear associated with transoesophageal echocardiography (TEE) and minimise potential discomfort, adverse reactions, and complications from the insertion of the oesophageal probe through the pharynx into the oesophagus, sedation is commonly used during TEE procedures. The conventional nasal cannula used during TEE in patients under deep sedation is limited to delivering oxygen flow rates in the range of 2–8 L/min [7]. Given that a substantial percentage of patients undergoing TEE are elderly and exhibit compromised cardiopulmonary function with diminished oxygen reserves, they tend to be more susceptible to anaesthetics and are at an increased risk of respiratory depression. This heightened vulnerability further increases the likelihood of perioperative hypoxaemia [8]. Consequently, ensuring optimal oxygenation throughout the examination is a critical challenge for anaesthesiologists.

A high-flow nasal cannula (HFNC) delivers warmed and humidified gas at a high flow rate and 100% oxygen concentration [9, 10]. A growing number of patients undergoing procedural sedation and analgesia depend on HFNC for oxygen delivery [11]. Given its proven capacity to enhance respiratory function and its excellent tolerability, HFNC has demonstrated efficacy in preventing hypoxia in a wide range of clinical scenarios. Randomised controlled trials have been conducted to validate HFNC in various procedural sedations, including gastrointestinal endoscopy, bronchoscopy, endoscopic retrograde cholangiopancreatography, and catheter ablation for atrial fibrillation [12–15]. Nay et al. applied HFNC for gastrointestinal endoscopy and demonstrated that it significantly lowers the risk of oxygen desaturation in patients at high risk of hypoxaemia during the procedure [16]. We hypothesized that HFNC could decrease the incidence of hypoxia in patients undergoing pre-procedural TEE for planned TEER with propofol sedation.

## Participants

Patients with moderate-to-severe MR who were scheduled to undergo propofol-sedated TEER were recruited. The inclusion criteria were as follows: (1) aged ≥ 18 years old, (2) BMI < 30 kg/m^2^, and (3) scheduled for mitral clip procedure. The exclusion criteria were as follows: (1) coagulation disorders or a tendency to nosebleeds, (2) high risk of reflux aspiration, (3) acute arrhythmia accompanied by haemodynamic instability, (4) sleep apnoea syndrome and (5) a history of difficult intubation.

### Randomization and blinding

Each patient was randomly allocated to either the HFNC group (Group H) or the nasal cannula oxygen group (Group C) in a 1:1 ratio using computer randomisation software (SPSS 21.0). The randomisation code was kept in a sealed, opaque envelope accessible only to the researchers and revealed immediately before anaesthesia induction. Postoperatively, the patients were interviewed by team members who were unaware of the randomisation group. Another team member recorded the participants’ responses and assigned them to groups based on the interviews.

### Interventions and anaesthesia

Prior to the procedure, the patients were instructed to fast overnight and continue fasting until TEE commenced. However, they were allowed to take dyclonine before TEE. After the patients entered the room, electrocardiography and peripheral oxygen saturation (SpO_2_) were continuously monitored during the procedure, and blood pressure was measured at a 2-minute interval. Patients in the nasal cannula group received 2 L/min of pure oxygen via nasal cannula, while those in the HFNC group received HFNC (Fisher & Paykel, Panmure, New Zealand) for oxygen inhalation (30 L/min, FiO_2_ = 100%). In both groups, the patients breathed calmly for 3 min, undergoing denitrogenation and oxygenation. Subsequently, an initial loading dose of propofol (1.0–1.5.0.5 mg/kg) was slowly injected intravenously. Once the eyelash reflex disappeared, the nasal cannula oxygen flow was increased to 6 L/min and the oxygen flow was adjusted to 60 L/min in the HFNC group. Oesophageal probe insertion was performed when the Ramsay score was > 4 (indicating a slow or no response to glabellar tapping or loud auditory stimulation). Continuous monitoring and maintenance of a Ramsay score > 4 were ensured throughout the examination. During the procedure, the infusion rate of propofol was maintained at 25–75 µg/kg/min and the total dosage was documented. Hypoxia in the periprocedural period was managed using the following measures: (1) discontinuing medication and arousing the patient, (2) lifting the jaw and increasing the flow rate of oxygen, (3) withdrawal of the endoscope and initiation of mask ventilation simultaneously, and (5) endotracheal intubation. In cases of hypotension identified during the evaluation, an intravenous injection of ephedrine at a dose of 5–10 mg was administered. Repeat dosing (cumulative maximum 30 mg) or transition to norepinephine 20 µg if no response within 2–3 min, with immediate procedure termination if instability persisted. If the heart rate fell below 50 beats/min, 0.25–0.50 mg atropine was administered.

### Outcomes and data collection

The primary outcome was the incidence of hypoxia (defined as 75% ≤ SpO_2_ < 90% for < 60 s) during anaesthesia [15, 17, 18]. The secondary outcomes were as follows: (1) subclinical respiratory depression (90% ≤ SpO_2_ < 95%) or severe hypoxia (SpO_2_ < 75% for any duration or 75% ≤ SpO_2_ < 90% for ≥ 60 s) [15]; (2) treatments for hypoxia; (3) adverse events related to HFNC within 30 min after the TEE procedure (nasal mucosal injury and bleeding, pneumothorax subcutaneous emphysema); (4) sedation-related adverse events (nausea/vomiting, reflux, sore throat, dyspnoea, airway obstruction, or choking) [19, 20]; (5) Hemodynamic parameters and procedural data during TEE, including mean heart rate, bradycardia, tachycardia, mean arterial pressure (MAP), lowest MAP, lowest systolic blood pressure, vasoactive medication use, propofol dosage, and examination time; and (6) doctors’ and patients’ satisfaction with the examination, which was also investigated using a 5-point Likert-type scale.

### Sample size

The estimated difference in the incidence of hypoxia between the two groups was used to calculate the sample size using PASS software (version 15.0; NCSS, LLC). According to our preliminary experimental results, we observed that 30% of the hypoxic events occurred during TEE. Based on studies comparing various oxygenation devices with standard oxygen therapy, we estimated that HFNC oxygen would reduce the incidence of desaturation from 30% to 15% [16]. With a power of 80%, an α of 5%, 1 degree of freedom, and accounting for a conservative attrition rate of 10%, our precise calculations determined that 267 patients would be required.

### Statistical analysis

Statistical analyses were performed using SPSS software (version 21.0; IBM SPSS, Chicago, IL, USA). Categorical variables are reported as counts with percentages (no. (%)), while numerical variables are expressed as mean ± standard deviation (SD) or median with ranges (interquartile range). For numerical variables, we employed an independent samples t-test (for normally distributed data) or Mann-Whitney U test (for non-normally distributed data), as appropriate based on Shapiro-Wilk testing. Fisher’s exact test was used to analyse categorical variables. No imputation was performed for missing data. For between-group differences, 95% confidence intervals (95% CIs) were calculated using the Newcombe method. Additionally, the relative risk (RR) and its exact 95% CI were computed. Statistical significance was set at *P* < 0.05.

## Results

**Fig. 1:**
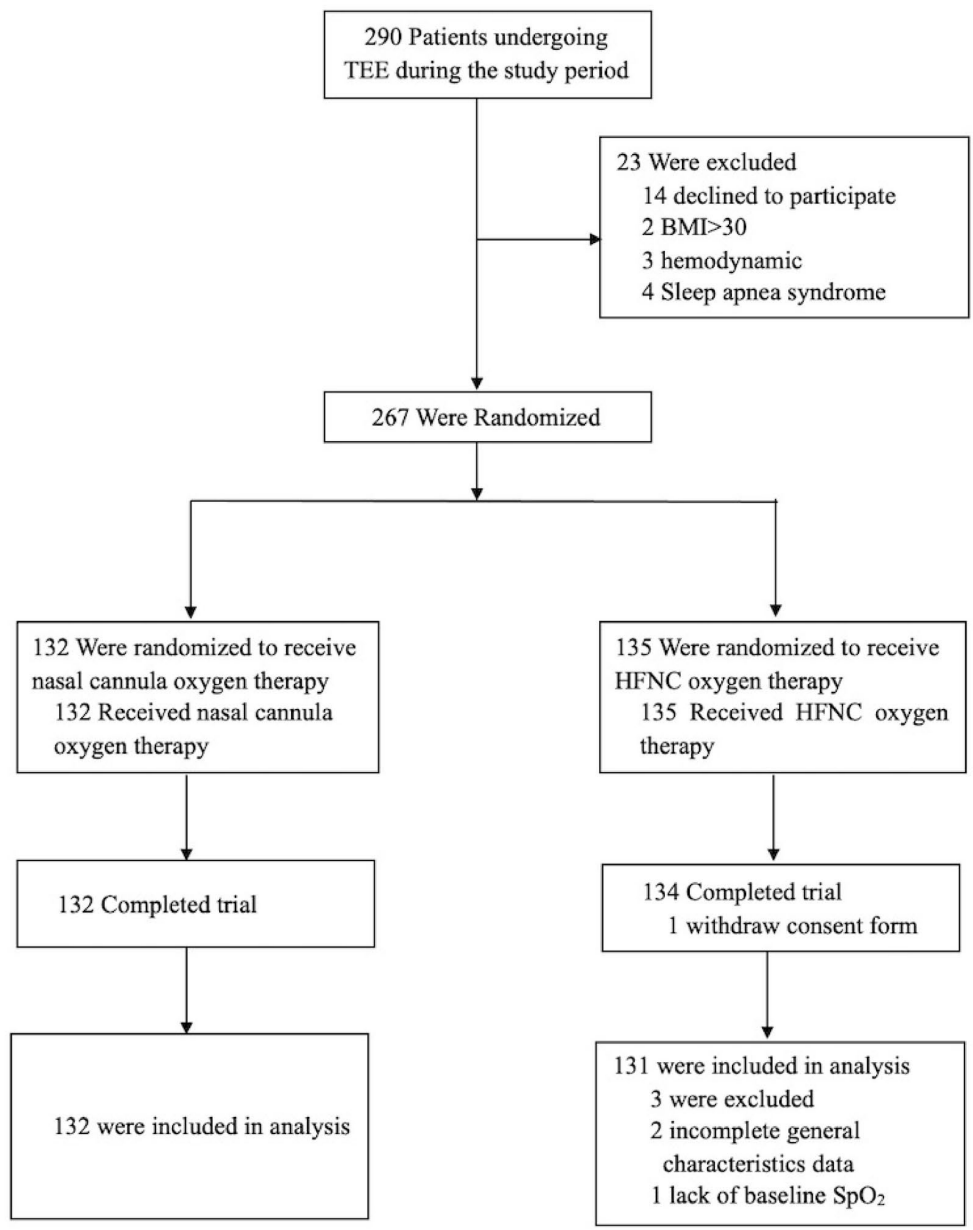
Study design flow diagram

February 2023 to January 2024, 290 patients were enrolled, of whom 23 were excluded (14 refused to participate in the study, 2 had BMI > 30; 3 had haemodynamic instability, and 4 had sleep apnoea syndrome). Finally, 267 patients were randomised into two groups. One patient in Group H was excluded because of withdrawal of consent, two because of incomplete general characteristic data, and one because of a lack of baseline SpO_2_ (Fig. 1). A comprehensive analysis was conducted on 263 patients. No statistically significant differences were found in the airway assessment indicators or baseline characteristics between the two groups (*P* > 0.1) (Table 1). The TEE time, propofol dosage, and haemodynamic indices did not differ significantly between the two groups (*P* > 0.1) (Table 2).

**Table 1:** Baseline characteristics of the study participants

Variables	Group C (*n* = 132)	Group H (*n* = 131)	*p* value
Age (ys)	71.73 ± 11.81	72.82 ± 10.68	0.497
Sex (male/female)	77/55	75/56	0.803
Body mass index (kg/m^2^)	22.36 ± 4.35	21.92 ± 4.13	0.835
ASA class (II/III)	34/98	39/92	0.468
Mallampati class (I/II/III/IV)	55/69/7/1	51/70/8/2	0.833
Mouth opening (1/2/3)	2/10/120	1/12/118	0.807
Thyromental distance(I/II/III)	120/12/0	118/13/0	0.837
History of hypertension	108 (81.82)	110 (83.97)	0.744
History of atrial fibrillation History of diabetes mellitus	45(34.09) 51(38.63)	43(32.82) 48 (36.64)	0.812 0.799
NYHA class (I/II/III/IV)	1/44/87/0	0/50/81/0	0.524
LV ejection fraction (%)	34.25 ± 6.11	33.34 ± 5.78	0.126
RV systolic pressure (mmHg)	44.32 ± 12.13	43.75 ± 11.93	0.930
Baseline SpO_2_ (%)	97 (97, 98)	97 (97, 98)	0.255

All data are expressed by mean ± standard deviations, median(interquartile range) or number (%). Abbreviations: ASA, American Society Anaesthesiologists. LV: left ventricle. Group C: nasal cannula oxygen group, Group H: HFNC group. Mouth opening (1/2/3): 1 = 1 finger, 2 = 2 fingers, 3 = 3 fingers; Thyromental distance (I/II/III): I > 6.5 cm, II 6–6.5 cm, III < 6 cm

**Table 2:** Hemodynamic parameters and procedural data during TEE

	Group C (*n* = 132)	Group H (*n* = 131)	*p* value
Duration, min	15.33 ± 3.22	16.11 ± 3.83	0.122
Dose of propofol, mg Mean heart rate, bpm Bradycardia Tachycardia	117.54 ± 12.50 72.52 ± 11.31 8(6.06) 12(9.09)	119.02 ± 11.81 71.82 ± 10.83 6(4.58) 10(7.63)	0.525 0.608 0.785 0.824
Hypotension (MAP < 60 mmHg)	35 (26.52)	32 (24.43)	0.243
Lowest mean arterial pressure			0.712
≥100-1 20 mmHg	6 (4.54)	5 (3.82)	
≥80 < 100 mmHg	37 (28.03)	39 (29.77)	
≥60–80 mmHg	55 (41.66)	56 (42.74)	
<60 mmHg	34 (25.76)	31 (23.66)	
Lowest systolic blood pressure			0.632
≥100–120 mmHg	51(38.64)	52 (39.69)	
≥ 80 < 100 mmHg	50 (37.88)	53 (40.46)	
≥60–80 mmHg	30 (22.73)	26 (19.85)	
<60 mmHg	1 (0.76)	0 (0)	
Vasoactive medication use	35 (26.52)	32 (24.43)	0.777

All data are expressed by mean ± standard deviations or number (%)). Continuous variables: Independent t-test or Mann-Whitney U test. Dichotomous variables: Chi-square or Fisher’s exact test. Ordinal categories (BP stratification): Mann-Whitney U test for overall distribution comparison; *P* represents test for difference in severity distribution between groups, not individual category comparisons

Alteration in vital signs (bradycardia, tachycardia) is defined as a change of > 25% from baseline

Compared with patients in Group C, those in Group H showed a significant reduction in the incidence of hypoxia, with rates decreasing from 23.48% to 13.74% (*P* = 0.040). The absolute risk reduction was 9.7% (95% CI: 0.4% to 19.1%), and the relative risk was 0.59 (95% CI: 0.35–0.99), indicating that HFNC use was associated with a 41% relative reduction in the risk of hypoxia compared with conventional nasal cannula.

Similarly, subclinical respiratory depression occurred significantly less frequently in Group H than in Group C (18.32% vs. 32.58%; *P* = 0.007), yielding an absolute risk reduction of 14.3% (95% CI: 3.9% to 24.6%) and a relative risk of 0.56 (95% CI: 0.36–0.87).

Regarding interventions for hypoxia, the requirement for jaw-lift manoeuvres was significantly lower in Group H (20.61% vs. 33.33%; *P* = 0.026), with an absolute risk reduction of 12.7% (95% CI: 2.1% to 23.3%) and a relative risk of 0.62 (95% CI: 0.41–0.94). Notably, no patients in Group H required mask ventilation (0% vs. 12.88% in Group C; *P* < 0.001), corresponding to an absolute risk reduction of 12.9% (95% CI: 7.2% to 18.6%).

One patient in Group C experienced respiratory arrest and required emergency tracheal intubation (0.76%), whereas no patients in Group H required mechanical ventilation. The absolute risk difference for mechanical ventilation was 0.8% (95% CI: − 0.7% to 2.2%), and the risk difference for severe hypoxia was 1.5% (95% CI: − 0.6% to 3.6%), though these differences did not reach statistical significance due to low event rates (Table 3).

**Table 3:** Efficacy outcomes

	Group C (*n* = 132)	Group H (*n* = 131)	Difference, % (95% CI)	Risk ratio (95% CI)	*p* value
Subclinical respiratory depression	43 (32.58)	24 (18.32)	9.7 (0.4 to 19.1)	0.59(0.35 to 0.99)	0.007
Hypoxia	31 (23.48)	18 (13.74)	14.3(3.9 to 24.6)	0.56(0.36 to 0.87)	0.040
Severe hypoxia	2 (1.51)	0 (0)	1.5(−0.6 to 3.6) ^†^	< 0.01(< 0.01 to 0.99)^‡^	0.498
Jaw lift	44 (33.33)	27 (20.61)	12.7(2.1 to 23.3)	0.62(0.41to 0.94)	0.026
Mask ventilation	17 (12.88)	0 (0)	12.9(7.2 to 18.6)	< 0.01(< 0.01 to 0.99)^‡^	0.000
Mechanical ventilation for endotracheal intubation	1 (0.76)	0 (0)	0.8(−0.7 to 2.2)	< 0.01(0.01 to 0.99) ^‡^	1.000

All data are expressed by number (%)

† Absolute risk difference with confidence interval crossing zero indicates no statistically significant difference

‡ Risk ratio calculated using continuity correction (0.5) due to zero events in Group H

The incidence of choking and paradoxical responses were lower in Group H than in Group C (*P* = 0.034) (Table 4). The satisfaction scores of the operators and anaesthesiologists in Group H were higher than those in Group C (operators, *P* = 0.003; anaesthesiologists, *P* = 0.000) (Table 5). After the patients were awakened, adverse events associated with HFNC breathing were observed at 5 and 30 min. However, none of the patients experienced nasal mucosal injury and bleeding, pneumothorax subcutaneous emphysema.

**Table 4:** Sedation-related adverse events

	Group C (*n* = 132)	Group H (*n* = 131)	*p* value
Paradoxical response^a^	15 (11.36)	5 (3.82)	0.034
Reflux	0 (0)	0 (0)	——
Nausea/vomit	0 (0)	0 (0)	——
Airway obstruction	0 (0)	0 (0)	
Choking	15(11.36)	5 (3.82)	0.034

All data are expressed by number (%)). N/A: Non applicable. ^a^Paradoxical response: Patients displayed unpredictable movement, overexcitement, and delirium after sedation with propofol

**Table 5:** Likert satisfaction scores of patients, operators and anesthesiologists

	Group C (*n* = 132)	Group H (*n* = 131)	*p* value
Patients	4(3, 4)	4 (4, 4)	0.088
Operators	4(4, 4)	4(4, 5)	0.003
Anesthesiologists	4 (3, 4)	4(4, 5)	0.000

All data are expressed by median (interquartile range)

## Discussion

In our study, HFNC reduced the incidence of hypoxia during TEE from 23.48% to 13.74% compared with nasal cannula oxygen. In Group C, one patient experienced respiratory arrest and required transfer to the cardiology intensive care unit for further treatment. Notably, Group H had no instances of severe hypoxia requiring tracheal intubation. The lower frequency of hypoxia in Group H allowed the surgeon to proceed with the procedure without delays caused by the need for anaesthesiological intervention, resulting in a more seamless operation. Consequently, both the surgeon and anaesthesiologist reported higher satisfaction levels in Group H. The incidence of hypoxia observed in this study was higher than that reported in the study by Kersey et al., which may be attributed to the fact that all patients in our study were complicated with moderate-to-severe MR and scheduled to undergo TEER [21]. These patients, characterised by a low ejection fraction and pulmonary hypertension, are likely to be more susceptible to hypoxia and haemodynamic instability.

We propose that HFNC effectively prevents hypoxia during sedated pre-procedural TEE for TEER planning via two primary mechanisms. First, HFNC generates positive airway pressure, thereby augmenting end-expiratory lung volume. The positive pressure in the pharynx during expiration, which is driven by a constant flow, is primarily influenced by the flow rate and the expiratory flow of the patient [22]. Positive nasopharyngeal pressure is essential to reduce upper airway obstruction and improve ventilation. The HFNC flow rate is positively correlated with nasopharyngeal pressure. At 50 L/min, the nasopharyngeal pressure can exceed 3 cmH_2_O [23]. In this study, an oxygen flow rate of 60 L/min was used, which is adequate to maintain SpO_2_.

Second, studies have demonstrated that HFNC maintains higher arterial oxygen partial pressure than conventional oxygen therapy, resulting in fewer hypoxic events [13, 15]. In our study, the HFNC group had an FiO_2_ set at 100%, and because of the high flow rate, the actual inspired oxygen concentration approached 100%. In contrast, the control group received 2–6 L/min via a standard nasal cannula, yielding an actual FiO_2_ of no greater than 40%. Clearly, a higher FiO_2_ is advantageous for maintaining SpO_2_.

By providing a continuous flow of fresh gas, HFNC can reduce intrapulmonary shunting, which is beneficial in patients with pulmonary congestion or oedema often observed in severe MR. MR can cause structural and functional cardiac impairments, leading to left atrial and left ventricular dilation. Additionally, pulmonary complications associated with MR may induce pulmonary hypertension and pulmonary congestion. While atrial fibrillation is common in this population and reflects MR disease severity, the balanced distribution between groups and hemodynamic stability at baseline ensured that rhythm status did not confound the primary outcome of procedural hypoxia. The physiological benefits of HFNC in the context of TEE with propofol sedation are multifaceted, though distinct from those observed in closed-mouth scenarios. We acknowledge that, unlike conventional assumptions regarding PEEP generation, the open-mouth requirement inherent to TEE probe insertion substantially attenuates the positive end-expiratory pressure effect. Recent measurements indicate that even at 50 L/min, HFNC produces only approximately 1.73 ± 0.82 cmH₂O of airway pressure when the mouth is kept open—a level insufficient to achieve meaningful alveolar recruitment or functional residual capacity expansion [24]. Additionally, the marginal positive pressure generated (1–2 cmH₂O), while insufficient for alveolar recruitment, may still contribute to reducing intrathoracic blood volume by promoting partial collapse of the inferior vena cava during inspiration, thereby decreasing preload—a mechanism previously described in heart failure patients [25]. This hemodynamic modulation, combined with reduced respiratory load and improved gas exchange through precise oxygen delivery, renders HFNC particularly beneficial for patients with cardiogenic pulmonary oedema [26, 27]. Our findings, demonstrating reduced hypoxia during sedated TEE, are consistent with these flow-dependent physiological principles and align with previous studies examining HFNC in procedural sedation contexts [25–29].

The study results further demonstrated lower incidence of choking (3.82% vs. 11.36%) and paradoxical responses (3.82% vs. 11.36%) in the HFNC group likely reflects two mechanisms. First, theheated, humidified gas delivered by HFNC (37 °C, 100% relative humidity) prevents drying of the oropharyngeal mucosa and reduces laryngeal irritability, thereby attenuating the gag and cough reflexes triggered by TEE probe insertion. Second, improved oxygenation and CO₂ clearance via pharyngeal dead space washout reduces cerebral hypoxia and hypercapnia-induced agitation, which are known triggers for paradoxical reactions (unpredictable movement, delirium) during propofol sedation. The continuous flow may also provide a subtle “airway splinting” effect that reduces the sensation of airway compromise.

### Generalizability and limitations

Our findings are most directly generalizable to adult patients with moderate-to-severe mitral regurgitation undergoing pre-procedural TEE for TEER planning under propofol-based deep sedation. The physiological profile (reduced ejection fraction, pulmonary hypertension) represents a high-risk cohort distinct from healthy outpatients undergoing diagnostic TEE.

This study has several limitations: (1) Single-center design: Conducted at a high-volume cardiac center with experienced operators; results may vary in centers with different sedation protocols. (2): Exclusion criteria: We excluded patients with BMI > 30 kg/m², severe baseline hypoxemia (SpO₂ <90% on room air), hemodynamic instability requiring ventilation, or anticipated difficult airways. Consequently, findings should not be generalized to morbidly obese patients, those with acute respiratory failure, or known difficult airways.

## Conclusions

HFNC reduces the incidence of hypoxia during pre-procedural TEE in patients with MR scheduled for TEER.

## Data Availability

The datasets used and/or analyzed during the current study are available from the corresponding author upon reasonable request.

## Abbreviations

ASA: American Society of Anesthesiologists
CONSORT: Consolidated Standards of Reporting Trials
HFNC: High flow nasal cannula oxygen therapy
NHYA: New York Heart Association
LV: Left ventricle
RV: Right ventricle
MAP: Mean arterial pressure
MR: Mitral regurgitation
SD: Standard deviation
SpO_2_: Peripheral oxygen saturation
TEE: Transesophageal echocardiography
